# Investigating the spatial distribution and effects of nearshore topography on *Acropora cervicornis* abundance in Southeast Florida

**DOI:** 10.7717/peerj.2473

**Published:** 2016-09-28

**Authors:** Nicole L. D’Antonio, David S. Gilliam, Brian K. Walker

**Affiliations:** Halmos College of Natural Sciences and Oceanography, Nova Southeastern University, Dania Beach, Florida, United States

**Keywords:** *Acropora cervicornis*, Distribution, GIS, Southeast Florida, Topography, Abundance

## Abstract

Dense *Acropora cervicornis* aggregations, or patches, have been documented within nearshore habitats in Southeast Florida (SE FL) despite close proximity to numerous anthropogenic stressors and subjection to frequent natural disturbance events. Limited information has been published concerning the distribution and abundance of *A. cervicornis* outside of these known dense patches. The first goal of this study was to conduct a spatially extensive and inclusive survey (9.78 km^2^) to determine whether *A. cervicornis* distribution in the nearshore habitat of SE FL was spatially uniform or clustered. The second goal was to investigate potential relationships between broad-scale seafloor topography and *A. cervicornis* abundance using high resolution bathymetric data. *Acropora cervicornis* was distributed throughout the study area, and the Getis-Ord Gi* statistic and Anselin Local Moran’s I spatial cluster analysis showed significant clustering along topographic features termed ridge crests. Significant clustering was further supported by the inverse distance weighted surface model. Ordinal logistic regression indicated 1) as distance from a ridge increases, odds of reduced *A. cervicornis* abundance increases; 2) as topographic elevation increases, odds of increased abundance increases; and 3) as mean depth increases, odds of increased abundance increases. This study provides detailed information on *A. cervicornis* distribution and abundance at a regional scale and supports modeling its distributions in similar habitats elsewhere throughout the western Atlantic and Caribbean. *Acropora cervicornis* is frequently observed and in areas an abundant species within the nearshore habitat along the SE FL portion of the Florida Reef Tract (FRT). This study provides a better understanding of local habitat associations thus facilitating appropriate management of the nearshore environment and species conservation. The portion of the FRT between Hillsboro and Port Everglades inlets should be considered for increased management and protection to reduce local stressors.

## Introduction

Dating back to the late Pleistocene, *Acropora cervicornis* has been an important component of southwestern Atlantic and Caribbean reef communities by providing habitat for numerous organisms and constructing a structurally complex reef framework that cannot be replaced by other corals (Aronson & Precht, 2001a; Bruckner et al., 2002; Knowlton, Lang & Keller, 1990). *Acropora cervicornis* has historically flourished over a wide range of reef zones, depths and hydrodynamic regimes. After decades of reported disturbances resulting in *Acropora cervicornis* population declines, this species was formally listed as threatened by the US Endangered Species Act in 2006. Numerous studies have investigated potential causes of *A. cervicornis* population decline in the southwestern Atlantic and Caribbean. Major contributors to this mortality include disease, predation, sedimentation, nutrient loading, thermal stress and storm damage (Aronson & Precht, 2001a; Bruckner et al., 2002; Davis, 1982; Gladfelter, 1982; Hoegh-Guldberg, 1999; Lang et al., 1992; Lessios, 1988; Lirman et al., 2011; Lirman & Fong, 1996; Porter, Battey & Smith, 1982; Porter et al., 2001; Roberts et al., 1982). These stressors, combined with the species dependence on asexual reproduction and limited potential for larval recruitment, make substantial recovery of populations’ uncertain (Aronson & Precht, 2001b; Hughes, Ayre & Connell, 1992; Knowlton, Lang & Keller, 1990).

A few remnant *A. cervicornis* populations have been reported in Florida (Vargas-Ángel, Thomas & Hoke, 2003; Williams, Miller & Kramer, 2008; Walker et al., 2012); Punta Rusia, Dominican Republic (Lirman et al., 2010); and Roatan, Honduras (Keck et al., 2005). The relative health of the populations described by Lirman et al. (2010) and Keck et al. (2005) may be attributed to the limited anthropogenic stress, either through separation from human influence by strong currents or located within a marine protected area offshore a region with limited development and small population. In Southeast Florida (SE FL), little distance exists between *A. cervicornis* populations and the effects of extensive urbanization from over population in addition to high frequency occurrences of natural disturbances such as tropical storms and hurricanes. Despite these anthropogenic and natural impacts which greatly stress the reefs along the northern extent of the Florida Reef Tract (FRT), several high density *A. cervicornis* thickets, or patches, have been documented nearshore SE FL at the latitudinal limit of the species. Some research has been completed on the distribution, abundance and dynamics of a few known patches in SE FL (Gilliam et al., 2015; Thomas, Dodge & Gilliam, 2000; Vargas-Ángel, Thomas & Hoke, 2003; Vargas-Ángel et al., 2006; Walker et al., 2012; Walker & Klug, 2014); however, little information has been published concerning *A. cervicornis* population characteristics outside of dense patch boundaries. Wirt et al. (2013) found 17% of *A. cervicornis* observations in Florida were in habitats not previously thought to be potential *Acropora* spp. habitat. These results indicate that there is a wide range of variable habitat where the species can survive which should not be neglected when investigating distribution. Furthermore, the recent discovery of 28 more large dense patches of *A. cervicornis* in SE FL via aerial imagery demonstrates the need for a regional understanding of *A. cervicornis* distribution, demographics, and status (Walker & Klug, 2014).

The first objective of this study was to conduct a spatially extensive and inclusive survey (9.78 km^2^) to determine whether *A. cervicornis* distribution in the nearshore habitat of SE FL was spatially uniform or clustered. The second objective was to investigate potential relationships between topography and *A. cervicornis* abundance using high resolution bathymetric data. The results of this study provide detailed information on *A. cervicornis* distribution at a regional scale, supporting and expanding previous knowledge. These data provide a better understanding of local habitat associations, address current gaps in baseline data, and support modeling *A. cervicornis* distributions in similar habitats in other locations throughout the western Atlantic and Caribbean thus facilitating appropriate management of the nearshore environment and species conservation.

## Methods

### Study area

**Figure 1:**
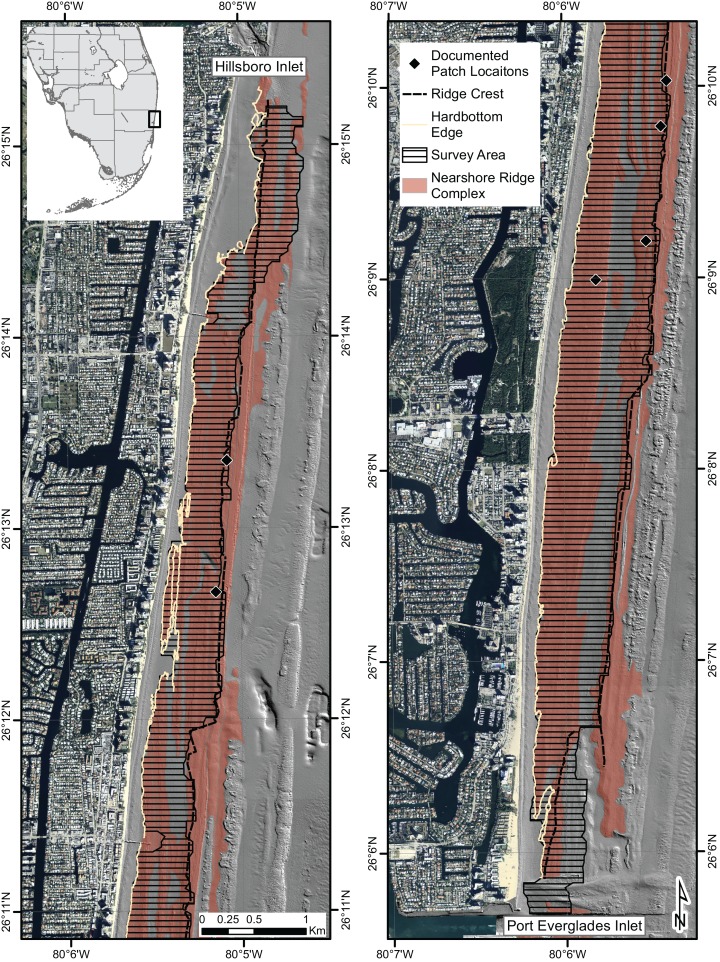
Survey area. Map showing location of eastern ridge crest, western hardbottom edge, and the portion of the NRC that was surveyed during this study. Locations of previously documented patches of *A. cervicornis* are also shown. Areas within the study area that are not shaded brown represent sand.

The reef habitats offshore SE FL (Miami-Dade, Broward, Palm Beach and Martin Counties) define the northern portion of the FRT (Banks et al., 2007). Nine major reef habitats have been described in this region including a nearshore ridge complex (NRC) (Walker, 2012). This study was conducted within a portion of the NRC offshore Broward County between Hillsboro and Port Everglades inlets (26.09–26.25 °N) (Fig. 1). The NRC in this area is within 1.2 km of the shoreline and is composed of ridges and areas of colonized pavement, rubble, and sand with depths ranging from three to six meters (Walker, 2012). Ridges have vertical relief from crest to base ranging from one to three meters.

### Sampling–distribution

Because our goal was to provide a more thorough description of *A. cervicornis* distribution within an area where it is already known to exist, we restricted our study to a portion of the NRC. The survey area was established from the western most hardbottom edge to the eastern most ridge crest within the NRC, spanning up to 900 m; an area where high densities of *A. cervicornis* have been reported (Gilliam et al., 2015; Thomas, Dodge & Gilliam, 2000; Vargas-Ángel, Thomas & Hoke, 2003; Vargas-Ángel et al., 2006; Walker et al., 2012; Walker & Klug, 2014). In the most northern and southern portions of the study area, the surveys were extended east, past the ridge crest due to the lack of hard bottom on the western side.

Surveys were conducted over a two-year period and completed in 2013 with the intent to characterize the relative nearshore abundance and distribution of *A. cervicornis* along 18 km of coastline between Port Everglades and Hillsboro inlets (Fig. 1). A search pattern and data collection procedure was adapted from the NOAA Recommended Survey Protocol for *Acropora* spp. (NOAA, 2007) and conducted at 1,956 sites. Sites were established by creating a 100 × 50 m polygon grid in ArcGIS covering the survey area. XY data was added to intersecting grid nodes to establish GPS points for each survey site then shifted so that each western-most site was 50 m east of the nearshore hard bottom edge. In the field, a buoy was dropped at the center of two 5,000 m^2^ sites. Divers on SCUBA attached 50 m tapes to the weighted buoy and swam with their tapes in opposite directions, either directly east or west. Divers counted the number of kicks to roll the entire 50 m tape out in order to estimate distance to swim away from the tape in a perpendicular direction, directly north or south. Divers surveyed sites in a parallel-track type pattern.

The objective of this study was to evaluate the current distribution and abundance of *A. cervicornis*. All *A. cervicornis* colonies regardless of size and state (attached, loose, or fragment) were included in the study. Although published studies investigating the fecundity of *A. cervicornis* fragments are limited, Lirman (2000) found that large fragments of *A. palmata* did, in fact, contain gametes. If fragments of *A. cervicornis* also contain gametes and are potentially reproductively viable, they ought to be included when estimating distribution and abundance. Therefore, it was important to include loose colonies and fragments with living tissue in addition to those attached to substrate. From more than six years of demographic monitoring data (E. Larson & D. Gilliam, 2013, unpublished data) conducted within the study area, the average single colony within the region has approximate dimensions of 30 × 30 cm for an average projected area a 900 cm^2^ using a square approximation. This approximation was used to convert the estimated area covered by large masses or thickets into the equivalent number of average sized colonies for the region. Divers recorded abundance within each site. The time it took to complete each survey ranged between 20–30 min depending on the abundance of *A. cervicornis* present at the site. To effectively and efficiently survey a large area which included a large number of sites, colonies were individually counted up to 50, and when abundances exceeded 50, they were placed into bins of 51–150 or > 150. In order to evaluate *A. cervicornis* association with substrate types within the study area, percentage of sand, rubble, and hardbottom was estimated for each site.

During October 2012, Tropical Storm Sandy traveled north through the Caribbean and the Bahamas; persistent northerly winds and the slow movement of Sandy caused large swells for the region along the east-central and southeastern coast of Florida. In addition to this and a gap in sampling periods, following established guidelines for sampling finite populations (Yamane, 1973; Israel, 1992), a subset of 292 sites were re-surveyed in 2013. Subset data from 2013 were compared to that from 2011 with a nominal logistic regression analysis using presence and absence data followed by Poisson regression analysis using count data. Results verified there was no significant change in presence or abundance of *A. cervicornis* between sampling periods (p < 0.05).

Data were fit to an inverse distance weighted interpolation (IDW) model to visualize continuous abundances throughout the area surveyed. IDW uses the measured values surrounding the prediction location to predict abundance for any unsampled location based on the assumption that objects that are close to one another are more similar than those that are farther apart. A benthic habitat layer (Walker, 2012) was used to mask the interpolation model so that only abundances within the NRC were predicted. The IDW was then clipped to the grid of 1,956 polygons, representing the area of each site.

A Getis-Ord Gi* statistic, or “hot spot” analysis, was performed in ArcGIS to locate spatial clusters of high and low abundances by calculating a Z-score and p-value. The Z-scores and p-values determine whether the spatial arrangement of high and low abundance was different than those expected in a random distribution (Getis & Ord, 1992). High Z-scores (> 1.65) and low p-values (< 0.05) indicated the spatial clustering of high abundance sites. Low Z-scores (< −1.65) and low p-values (p < 0.05) indicated spatial clustering of low abundance sites. Z-scores near zero indicated no apparent spatial clustering.

The Anselin Local Moran’s I spatial cluster and outlier analysis was also performed. In addition to calculating Z-scores and p-values to determine statistically significant clustering like the Gi* statistic, this analysis also identified spatial outliers (high abundance sites surrounded by low abundance sites and vice versa) (Anselin, 1995). The output was displayed to distinguish between a statistically significant (p-value < 0.05) cluster of high abundance (HH), cluster of low abundance (LL), and outliers that either had a high abundance surrounded by low abundance (HL) or had a low abundance surrounded by high abundance (LH).

### Sampling–topographic metrics

A LIDAR bathymetric survey was conducted during 2007 which covered an approximate total area of 110 km^2^ including this entire study survey area (Ramsay & Sinclair, 2008). High resolution (four meter) bathymetry points from the LIDAR data set were imported as XYZ data. Triangulated irregular networks (TINs) were created from the LIDAR data points to produce a three-dimensional surface and used to calculate topographic metrics (Jenness, 2004; Walker, 2008; Wang & Lo, 1999). The TINs referenced the grid of 1,956 polygons, representing each site. The TIN analysis was performed using the Surface Tools Extension (Jenness, 2008) in ArcGIS to calculate a set of topographic metrics within each individual site. Calculated metrics included surface rugosity, elevation change (maximum depth–minimum depth), and mean depth. The surface rugosity index was calculated by dividing the three-dimensional surface area by the two-dimensional planar area. Additionally, the distance was measured from the geometric center of each grid polygon to the closest prominent ridge crest. Site to ridge proximities were measured by drawing of a vector line over the shallowest points along the ridges in the 2007 bathymetry.

**Figure 2:**
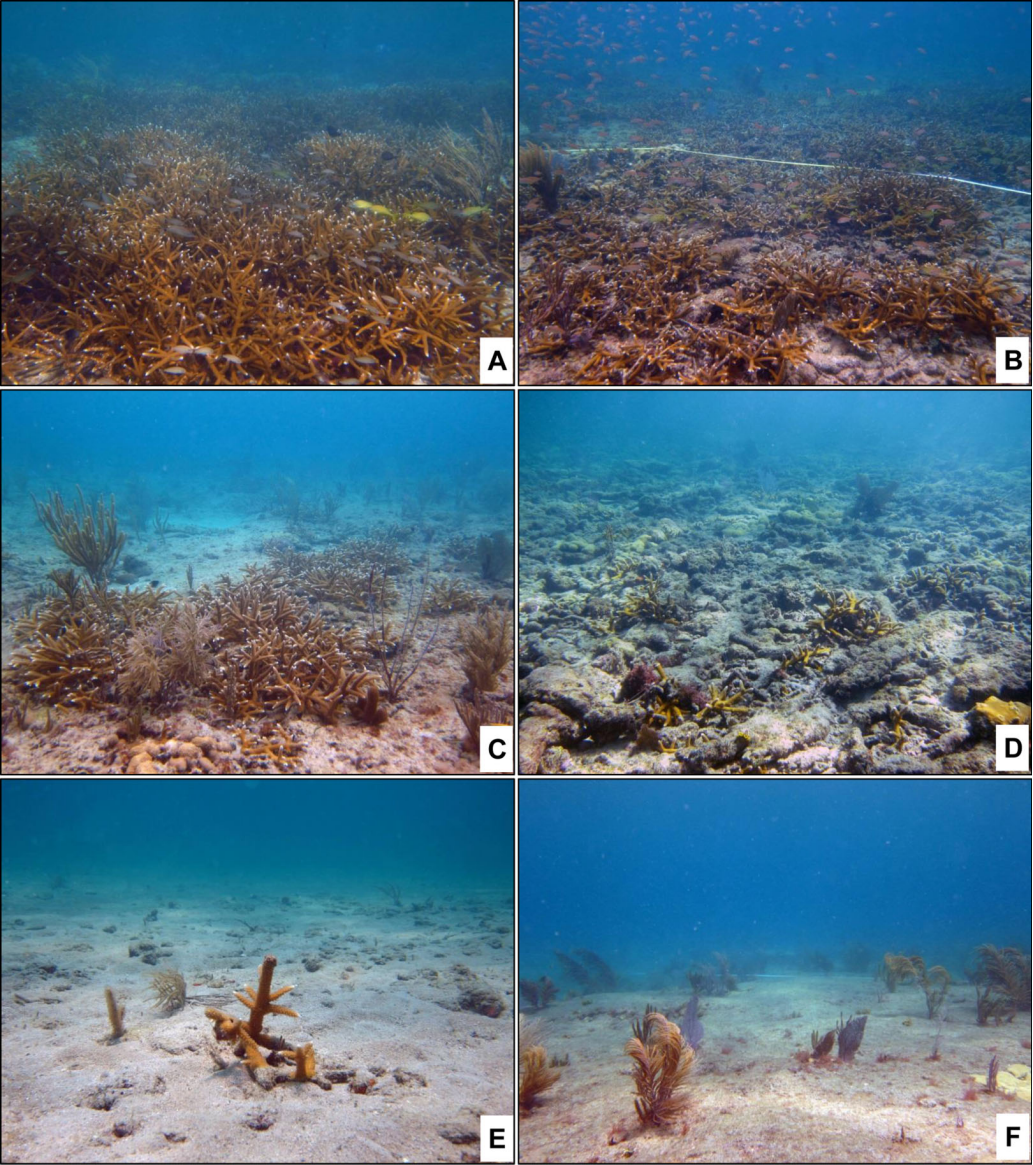
Illustrated examples of sites classified within each abundance category. (A) Site recorded in the Abundant category (> 150 colonies/5,000 m^2^), (B) a site recoded in the Common abundance category (51–150 colonies/5,000 m^2^), (C) a site recorded in the Frequent abundance category (31–50 colonies/5,000 m^2^), (D) a site recorded in the Occasional abundance category (11–30 colonies/5,000 m^2^), (E) a site recorded in the Rare abundance category (1–10 colonies/5,000 m^2^), and (F) a site in the Absent abundance category.

*Acropora cervicornis* abundances recorded during the site surveys were placed into one of six bins using a 6-point ACFORA scale: Abundant (species is prevalent within the site, > 150 occurrences), Common (species is encountered often, with more than 50 occurrences but less than 150), Frequent (species is present, more than 30 occurrences but less than 51), Occasional (present in low numbers, with more than 10 occurrences but less than 31), Rare (10 or less occurrences of the species at the site), and Absent (no occurrences of the species at the site) (Fig. 2; Table 1). Abundance categories, dominant substrate, and topographic metrics were analyzed using a mixed-effects ordinal logistic regression model.

**Table 1:** *Acropora cervicornis* abundance categories and number of corresponding sites. Colony abundance categories used to compare *A. cervicornis* abundance to topographic metrics and substrate type in ordinal logistic regression analyses, and the number of survey sites represented by each category.

Abundance	Category		# of sites
0	Absent	No occurrences of the species at the site	1,016
1–10	Rare	10 or less occurrences of the species at the site	250
11–30	Occasional	Present in low numbers	151
31–50	Frequent	Species is present in moderate numbers	100
51–150	Common	Species is encountered often	217
> 150	Abundant	Species is prevalent within the site	222

## Results

### Overall distribution

Surveys were conducted February through November 2011 and May through October 2013. Nearly half of all 1,956 survey sites had the presence of *A. cervicornis* (940 sites; 48%). *Acropora cervicornis* was rare at 13% of the sites, occasional at 8% of sites, frequent at 5% of sites, common at 11% of sites, and abundant at 11% of sites (Table 1). Nearly half of the 1,956 sites surveyed (48%) were characterized as hardbottom-dominated, 17% of all sites were rubble-dominated, 30% were sand-dominated, 1% was dominated equally by hardbottom and rubble, 3% were dominated equally by hardbottom and sand, and 1% was dominated equally by rubble and sand. *Acropora cervicornis* was more frequently present at sites dominated by hardbottom (26%) and rubble (12%). A summary of sites with the presence of *A. cervicornis* on each substrate type is shown in Table 2.

**Table 2:** Abundance and dominant substrate results. Number of sites in each abundance category by dominant substrate. Dominant substrate types included hardbottom (HB), rubble (RBL), sand (SND), hardbottom and rubble (HB & RBL), hardbottom and sand (HB & SND), rubble and sand (RBL & SND). Substrate composition estimates were not recorded for 12 of 1,956 sites.

Category	HB	RBL	SND	HB & RBL	HB & SND	RBL & SND	Missing data	Total
Absent	419	93	450	5	42	6	1	1,016
Rare	106	56	74	4	5	1	4	250
Occasional	82	35	29	2	2	0	1	151
Frequent	63	19	17	0	0	1	0	100
Common	137	62	9	3	1	2	3	217
Abundant	127	71	10	6	3	1	4	222
Total	934	336	589	20	53	11	12	1,956

**Figure 3:**
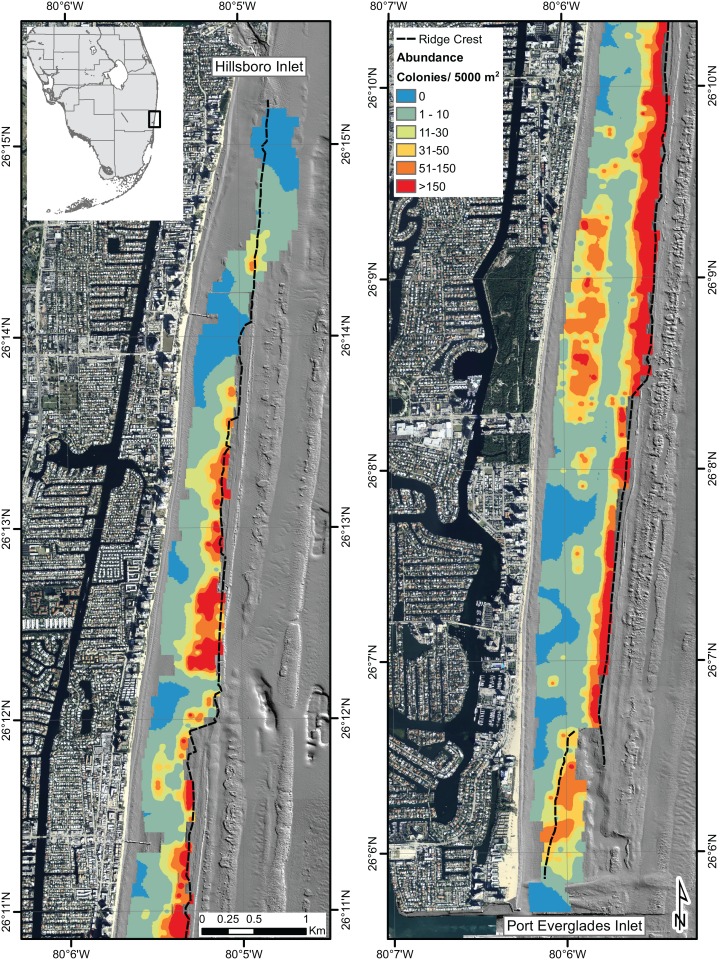
Inverse distance weighted interpolation model of *Acropora cervicornis* abundance within surveyed area. Map showing the inverse distance weighted interpolation model, illustrating the abundances of *A. cervicornis* observed within the study area, unobstructed by survey site boundaries.

**Figure 4:**
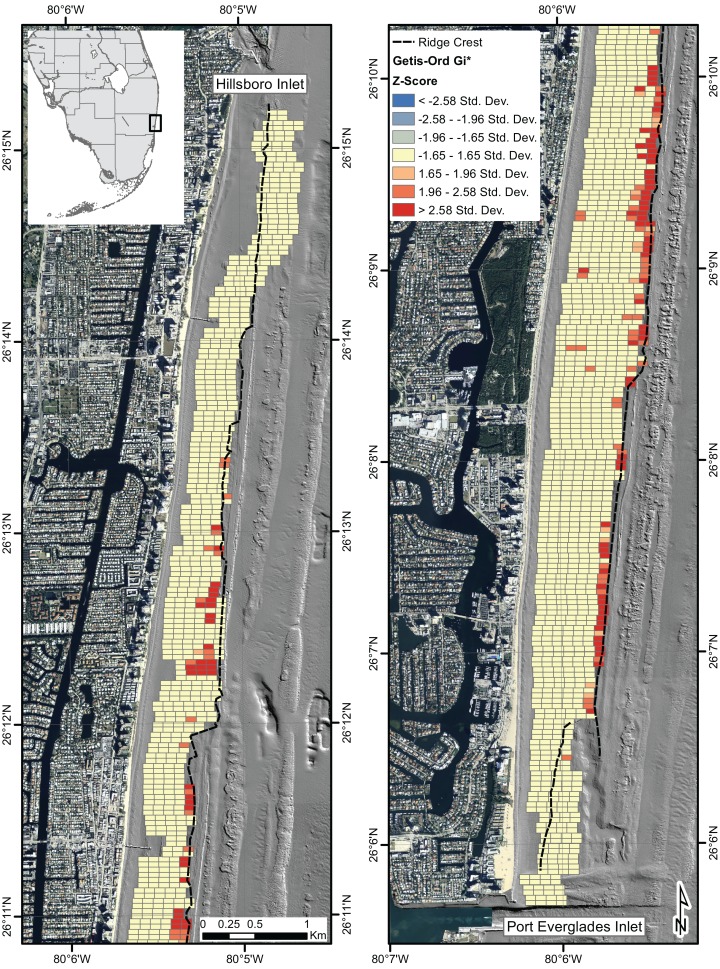
Results of Getis-Ord Gi* statistic. The Getis-Ord Gi* statistic shows significantly high abundances of *A. cervicornis* clustering along the eastern ridge crest.

The IDW interpolation illustrates the results of *A. cervicornis* abundance observed throughout the survey area unobstructed by site boundaries (Fig. 3). The Getis-Ord Gi* statistic (Fig. 4) shows statistically significant (p-value < 0.05) high abundance clustering of *A. cervicornis* along nearly the entire eastern ridge crest of the NRC as well as a several surveyed areas associated with shoreward ridges. The Anselin Local Moran’s I also shows clustering in the same locations and no outliers.

### Topography and abundance

A mixed-effects ordinal logistic regression with robust clustered standard errors around substrate was conducted. The fixed effects were distance to closest ridge, surface rugosity, elevation, and mean depth. All of the predictor variables showed statistical significance in the ordinal logistic regression model (p < 0.05) (Table 3). Results showed the following: (1) when the distance to a ridge increases by one meter, the odds of a site moving from a higher abundance category to a lower category are 1.08 times greater; (2) when elevation change increases by one meter, the odds of moving from a lower abundance category to a higher category are 1.25 times greater; and (3) when mean depth increases by one meter, the odds of moving from a lower abundance category to a higher category are 1.27 times greater. Surface rugosity showed statistical significance, however, the odds of a change in abundance category were so low that the predictor can be considered applicably insignificant. This result can likely be attributed to the calculation of rugosity at the coarse scale of 100 × 50 m (area of each site).

**Table 3:** Results of ordinal logistic regression analysis. Results of ordinal logistic regression model to predict *A. cervicornis* abundance category by distance to a ridge, surface rugosity, elevation change, and mean depth.

Predictor	*χ*^2^	Std. error	*P*	Odds ratio
Distance to the closest ridge	−0.007	0.001	< 0.0001	0.923
Surface rugosity	−0.045	0.059	< 0.0001	3.25 × 10^−197^
Elevation (max depth–min depth)	0.222	0.048	< 0.0001	1.249
Mean depth	0.242	0.034	< 0.0001	1.274

## Discussion

This is the largest published area (9.78 km^2^) surveyed in such detail to document *A. cervicornis* abundance and distribution. Although the nearshore area between Port Everglades and Hillsboro Inlet is a small segment of the FRT that is greatly impacted by anthropogenic stressors, the Threatened species, *A. cervicornis*, is frequently observed and, in areas, an abundant species within this nearshore habitat.

All of the surveys occurred on the NRC (Walker, 2012) which is characterized as sporadic sandy areas intermixed with consolidated hardbottom. This was confirmed by 66% of all survey sites characterized as having hardbottom, rubble, or both as the dominant substrate types. The observed high abundances of *A. cervicornis* on rubble were not expected as the presence of loose substrate potentially increases the chances of physical damage to or burial of existing colonies. Lirman & Fong (1997) stated that a critical factor affecting ability of a fragment to successfully attach is substrate type and that in a study conducted in Florida, *A. palmata* fragments that landed on rubble substrate had higher survivorship and lower percent tissue loss than those deposited on relict reef substrate. Bak & Criens (1981) found that *A. palmata* fragments fuse to other living fragments faster than to any other type of substrate. Fusion of *A. cervicornis* fragments may be occurring in rubble-dominated areas away from prominent ridges in SE FL (Fig. 2B). The species’ potential to persist in rubble, as shown in this study, is consistent with high survivorship of *A. palmata* fragments on rubble and hardbottoms over a four-month period (Lirman, 2000) and the fusing together of *A. cervicornis* fragments in soft or sandy bottoms (Highsmith, 1982).

The significant spatial clustering of areas with abundant *A. cervicornis* spanned over 12 km along a clearly-defined unique topographic ridge. Klug (2015) assessed the benthic cover of five cross-shelf corridors within the northern portion of the FRT (25.5–26.3 °N) and found over 85% of all *A. cervicornis* colonies observed were within just two corridors, both of which were included in this study. In addition to the seven patches noted by Vargas-Ángel, Thomas & Hoke (2003) in SE FL, Walker & Klug (2014) recently identified 28 high density *A. cervicornis* patches not previously reported. The spatial clustering observed in our study supports the existence of these high density patches associated with the western side of the ridge crest. Vargas-Ángel, Thomas & Hoke (2003) stated that the highest density thickets occurred in calmer waters closer to shore. This includes the two monitored patches that were documented to have declined in cover between 2008 and 2011 (Walker et al., 2012). In contrast to the results of the current study, Vargas-Ángel, Thomas & Hoke (2003) reported the thickets that occurred on the carbonate coquina platform (i.e. ridge crest), were composed of interspersed colonies of *A. cervicornis* with patches of hard bottom and isolated gorgonians. While this was observed on the eastern-most side of the ridge crest, on the western edge, large and continuous areas of *A. cervicornis* were noted. It appears that wave energy at the crest promotes colony fragmentation and fragment movement to the west. Wave energy dissipates enough for fragments to attach and flourish on the leeward side of the crest. The topographic analysis supported this by the increased odds for higher abundances with an increase in elevation change. Larger changes in elevation were most often observed at sites that included the ridge crest, where clustering of high abundances also occurred. The increased odds for higher abundances as depth increases may be explained by many sites exhibiting both high elevation changes and high *A. cervicornis* abundance, not necessarily the presence of higher abundances observed at greater depths.

This study shows that nearshore bottom topography can provide some insight into predicting the distribution of high abundances of *A. cervicornis* but alone, it cannot explain where and why high and low densities of *A. cervicornis* exist. This is a unique coral in that, to some extent, it is motile. Two patches of *A. cervicornis* in SE FL were found to move in a northwest direction expanding in area over a three year monitoring period (Walker et al., 2012). This is an indicator of oceanographic influence on location of patch growth. The reported direction of patch movement can be expected since fragment transport mimics nearshore oceanographic processes (Lee & Mayer, 1977). These processes result in a dominant north and west direction of water movement and are strongly influenced by the Florida Current (Banks et al., 2008; Lee, Schott & Zantopp, 1985; Molinari & Leaman, 1987). It is clear there are key explanatory variables required to successfully predict *A. cervicornis* distribution that our study did not include. Many areas where *A. cervicornis* colonization has occurred are the result of the either sexual recruitment or the transport of fragments broken off colonies from other distant locations (Highsmith, 1982; Highsmith, Riggs & D’Antonio, 1980). In order to follow the transport of loose fragments to their point of attachment, elements of directional water movement must be incorporated into the research. This is an area of study for future exploration in order to more fully understand the forces acting upon the population of *A. cervicornis* nearshore in SE FL.

The directional movement of the patches studied by Walker et al. (2012) and the *A. cervicornis* population within this region may also be influenced by effects of climate change. There is some conjecture that *Acropora* communities in SE FL may be exhibiting poleward expansion as sea surface temperatures rise (Precht & Aronson, 2004; Walker, 2012). Walker (2012) defined three potential biogeographic barriers to northern expansion in SE FL, one of them being at the northern limit of our survey area, the Hillsboro boundary. The high abundances observed in our study are already located near the species’ present latitudinal margin and a lack of suitable nearshore substrate combined with more frequent and intense upwelling may be limiting any potential northern expansion (Walker, 2012; Walker & Gilliam, 2013).

This research supplements previous work done in SE FL to characterize abundance and distribution of *A. cervicornis*. In addition to supporting recent findings of several new *A. cervicornis* dense patches, results of this study illustrate the gradient of abundances that exist outside of these high density areas. This gradient may be key in answering questions proposed by Walker et al. (2012): is coral cover truly declining or are patches just spreading out and/or moving due to coastal water circulation processes and/or climate change? These findings offer a more thorough description of the distribution and abundance of the Threatened coral, *A. cervicornis,* than previously known while providing a better understanding of local habitat associations in SE FL. The portion of the FRT between Hillsboro and Port Everglades inlets should be considered for increased management and protection to reduce local stressors.

## Supplemental Information

10.7717/peerj.2473/supp-1 Supplemental Information 1 *A.cervicornis* Abundance and Topographic Metrics.

## Additional Information and Declarations

### Data Deposition

The following information was supplied regarding data availability: The raw data has been supplied as Supplemental Dataset Files.
